# Calcitriol as a Therapeutic Strategy for Ferric Carboxymaltose–Induced Hypophosphatemia

**DOI:** 10.7759/cureus.96256

**Published:** 2025-11-06

**Authors:** Freny Patel, Prince Modi, Bosky Modi, Rangesh Modi

**Affiliations:** 1 Internal Medicine, Pramukhswami Medical College, Karamsad, IND; 2 Endocrinology, Medical College of Wisconsin, Milwaukee, USA; 3 Gastroenterology and Hepatology, The University of Chicago Medicine, Chicago, USA

**Keywords:** 1.25- dihydroxy vitamin d, fgf 23, hypophosphatemia, intravenous iron supplement, s: hypocalcemia

## Abstract

We describe an 83-year-old man with idiopathic protein-losing enteropathy on chronic total parenteral nutrition who developed profound, persistent hypophosphatemia after intravenous ferric carboxymaltose (FCM) infusion for anemia. His course was notable for hypophosphatemia refractory to phosphate supplementation, accompanied by hypocalcemia, secondary hyperparathyroidism, suppressed 1,25-dihydroxy vitamin D, and markedly elevated fibroblast growth factor 23 (FGF23). These findings were consistent with FCM-induced hypophosphatemia, a condition increasingly recognized as part of the “6H Syndrome.” Initiation of oral calcitriol led to rapid normalization of calcium and phosphate levels and enabled long-term stability without further IV iron exposure. This case underscores the need for heightened clinical awareness of FCM-associated electrolyte disturbances, especially in patients with baseline malnutrition or malabsorptive disorders.

## Introduction

Iron deficiency is the most common nutritional deficiency worldwide, with higher prevalence among individuals with chronic illnesses, menstruating women, and patients with gastrointestinal disorders [1]. Oral iron supplementation remains the first-line therapy; however, its use is limited by the absorptive capacity of the human small intestine and gastrointestinal adverse effects (abdominal pain, constipation, nausea, or diarrhea). Modern IV iron formulations, including ferric carboxymaltose (FCM), ferric derisomaltose (FDI), and ferrumoxytol, allow for rapid and efficient repletion of iron stores, especially in malabsorptive disorders, without any gastrointestinal adverse effects [2,3]. While these preparations are generally well tolerated, adverse effects can occur, ranging from minor infusion reactions to more serious metabolic complications.

One such complication is IV iron-induced hypophosphatemia (IIH), which has been increasingly reported, particularly with FCM and, to a lesser extent, FDI [2-4]. IIH may present with fatigue, myalgia, bone pain, and muscle weakness, which can mimic ongoing iron deficiency anemia (IDA), and in severe cases can lead to respiratory compromise, osteomalacia, and fractures [5,6]. Despite growing recognition, the pathophysiology of IIH remains underappreciated in clinical practice. Current evidence points to a fibroblast growth factor 23 (FGF23)-mediated mechanism, resulting in renal phosphate wasting, hypovitaminosis D, hypocalcemia, and secondary hyperparathyroidism [2,5,6]. While hypophosphatemia may self-resolve over weeks to months after stopping IV iron, certain population may develop severe recurrent or prolonged hypophosphatemia requiring innovative management strategies such as use of oral 1,25 vitamin D [7].

## Case presentation

An 83-year-old man with a history of gastroesophageal reflux disease (GERD), iron deficiency anemia (IDA), hyperlipidemia, and idiopathic protein-losing enteropathy (PLE) dependent on total parenteral nutrition (TPN) was admitted for evaluation of severe asymptomatic hypophosphatemia. He had no history of surgery, smoking, alcohol, or illicit drug use. Medications included atorvastatin 40 mg, omeprazole 20 mg, calcium carbonate 1250 mg, and cholecalciferol 50 mcg (2000 IU) daily with no changes over the past year. He denied use of NSAIDs or herbal supplements. He had been on stable daily TPN for 10 years due to chronic malabsorption from idiopathic PLE with weekly lab monitoring. His TPN regimen (1500 mL over 16 hours/day) included: 250 g dextrose, 90 g protein, 50 g fat, standard vitamins/minerals, 65 mEq potassium, eight mEq magnesium, 10 mEq calcium, 40 mmol phosphate, 115 mEq sodium, 57 mEq chloride, and 58 mEq acetate.

Four weeks before admission, he received IV ferric carboxymaltose (FCM) for anemia. Two weeks later, routine labs showed severe hypophosphatemia (<1 mg/dL). Due to the risk of calcium-phosphate precipitation, additional phosphate could not be added to TPN. He received intermittent potassium phosphate supplementation, but phosphate levels remained low (1-1.5 mg/dL), prompting admission. On admission, he denied fatigue, muscle weakness, cramps, paresthesia, or shortness of breath. His diarrhea from PLE was at baseline two loose stools per day. He was hemodynamically stable and afebrile. His physical examination showed mild muscle wasting and was otherwise unremarkable.

His complete blood count (CBC) was normal except for chronic anemia with hemoglobin 11.2 g/dL, which was lower than his baseline of 13.0 g/dL. Serum iron studies before his recent iron infusion showed low iron, high total iron binding capacity (TIBC), low percent transferrin saturation, and low ferritin. His serum B12 and folate were normal. His glucose, renal, and liver function were normal. Total protein and albumin were low at 4.8 g/dL and 2.7 g/dL, respectively, although at his baseline due to PLE. His electrolytes were normal except for severe hypophosphatemia of <1 mg/dL and hypocalcemia with corrected calcium of 6.4 mg/dL. Urinalysis was normal, and the electrocardiogram showed a normal sinus rhythm. A CT scan of the abdomen and pelvis was unremarkable.

His parathyroid hormone (PTH) was high at 501 picogram/ml, while serum 25-hydroxy vitamin D, calcitonin, and PTH-related peptide were normal. After 48 hours of aggressive calcium and phosphate repletion, his serum corrected calcium remained low at 7.6 mg/dL and phosphate at 1.8 mg/dL. His 24-hour urine fractional phosphate excretion (FEphos) was high at 93% (normal <5%), and 24-hour urine fractional calcium excretion (FECa) was 1.4% (normal 1-2 %). His stool electrolyte panel was normal, especially phosphate of 33 mg/dL (abnormal >102). His urine fibroblast growth factor-23 (FGF-23) was elevated at 264 picogram/ml (normal <59), and serum 1,25 vitamin D was low at 15 picogram/ml (normal 18-64). Table 1 summarizes all laboratory values.

**Table 1:** Summary of Laboratory Values U: Unit; RU: Reference unit; mg: Milligram; ml: Milliliter; pg: Picogram; ng: Nanogram; pmol: Picomole; fL: Femtoliter; µg: Microgram; dL: Deciliter; g: Gram; mmol: Millimole; µL: Microliter.

Laboratory Type	Test (Blood/Urine)	Measured Value	Reference Range (SI Units)
Complete Blood Count (CBC)	White Blood Cell Count	7.1 ×10³/µL	3.5–11 ×10³/µL
	Hemoglobin	11.2 g/dL	13.5–17.5 g/dL
	Mean Corpuscular Volume	93.8 fL	80–100 fL
	Platelet Count	177 ×10³/µL	150–450 ×10³/µL
Nutritional Profile	Folate	10.2 ng/mL	4-26 ng/mL
	Vitamin B12	724 pg/mL	200–900 pg/mL
Iron Studies	Serum Iron	22 µg/dL	60–170 µg/dL
	Total Iron Binding Capacity	450 µg/dL	240–450 µg/dL
	Transferrin Saturation	13%	20–50%
	Ferritin	17 ng/mL	30–400 ng/mL
Inflammatory Marker	C-Reactive Protein	<3 mg/L	<10 mg/L
Metabolic Profile	Glucose	85 mg/dL	60–99 mg/dL
	Sodium	143 mmol/L	135–145 mmol/L
	Potassium	3.8 mmol/L	3.5–5.0 mmol/L
	Chloride	108 mmol/L	98–108 mmol/L
	Bicarbonate	23 mmol/L	22–29 mmol/L
Renal Function	Blood Urea Nitrogen	14 mg/dL	7–20 mg/dL
	Creatinine	0.77 mg/dL	0.5–1.4 mg/dL
Liver Function	Total Protein	4.8 g/dL	6.0–8.3 g/dL
	Albumin	2.7 g/dL	3.5–5.0 g/dL
	Total Bilirubin	1.0 mg/dL	0.3–1.2 mg/dL
	Aspartate Aminotransferase	30 U/L	8–37 U/L
	Alanine Aminotransferase	27 U/L	8–35 U/L
	Alkaline Phosphatase	125 U/L	45–120 U/L
Minerals	Magnesium	1.8 mg/dL	1.6–2.5 mg/dL
	Phosphate	<1 mg/dL	2.5–4.5 mg/dL
	Corrected Calcium	6.44 mg/dL	8.4–10.2 mg/dL
Hormonal Profile	Parathyroid Hormone-related peptide	0.5 pmol/L	<2 pmol/L
	25-Hydroxyvitamin D	28 ng/mL	30–100 ng/mL
	1,25-Hydroxyvitamin D	15 pg/mL	18-64 pg/mL
	Parathyroid Hormone	501 pg/mL	15–65 pg/mL
	Calcitonin	<5 pg/mL	<10 pg/mL
Urinary Studies	Urine Fibroblast Growth Factor-23	264 RU/mL	<180 RU/mL
	24-hr Urine Creatinine	866 mg/day	500–2000 mg/day
	24-hr Urine Calcium	102 mg/day	100–300 mg/day
	24-hr Urine Phosphate	1052 mg/day	400–1300 mg/day
	Fractional Excretion of Phosphate	93 %	<20 %
	Fractional Excretion of Calcium	1.4%	1-2 %

His chronic diarrhea was at baseline, and stool phosphate excretion was normal, hence, excluding worsening malabsorption as the etiology. He denied alcohol or antacid use, which can cause hypophosphatemia, and long-term stable TPN use excluded refeeding syndrome or malnutrition as etiology. He had evidence of hyperphosphaturia due to hyperparathyroidism, which was believed to be secondary due to hypocalcemia, as primary or tertiary hyperparathyroidism causes hypercalcemia. His serum vitamin D was normal, urine calcium excretion was normal, and he received adequate oral and IV calcium supplementation. His urine FGF 23 was high, which in turn inhibits renal one alpha hydroxylase activity and leads to reduced formation of 1,25 hydroxy vitamin D, which is the primary activated form of vitamin D. This was believed to be the cause of his hypocalcemia, as 1,25 vitamin D is responsible for intestinal and urine calcium reabsorption. Traditionally, elevated serum FGF-23 occurs in mesenchymal tumors, chronic kidney disease, and certain genetic disorders such as X-linked hypophosphatemia that manifest at a younger age.

Intravenous iron, especially FCM, is an important cause for FGF-23-mediated hypophosphatemia and remained the likely diagnosis in our case. Due to recurrent need for aggressive calcium and phosphate supplementation as well as the possibility that IV iron-mediated hypophosphatemia can last for more than 1-2 months, we initiated oral calcitriol at 0.25 microgram daily. After 48 hours of initiating calcitriol, his serum corrected calcium was 8.4 mg/dL, and serum phosphate was 2.9 mg/dL without additional IV replacement. At discharge, he continued to remain on the same TPN formula, oral calcium, and 25-hydroxy vitamin D in addition to oral calcitriol 0.25 microgram daily. An attempt to wean off calcitriol at 3 months led to the recurrence of hypocalcemia and hypophosphatemia.

At 6 months, he was successfully weaned off calcitriol without recurrence of electrolyte abnormalities. Table 2 shows the relevant laboratory trends following calcitriol treatment.

**Table 2:** Laboratory trend following calcitriol treatment mg: Milligram; dL: Deciliter; pg: Picogram; mL: Milliliter; ng: Nanogram; RU: Reference Units.

Test (Blood/Urine)	Pre-treatment labs	48 hours post-calcitriol	6 months post-calcitriol	Reference range (SI units)
Serum Phosphate	<1 mg/dL	2.9 mg/dL	3.1 mg/dL	2.5–4.5 mg/dL
Serum Corrected Calcium	6.44 mg/dL	8.4 mg/dL	8.6 mg/dL	8.4–10.2 mg/dL
Serum Parathyroid Hormone	501 pg/mL	208 pg/mL	45 pg/mL	15–65 pg/mL
25-Hydroxyvitamin D	28 ng/mL	N/A	38 ng/mL	30–100 ng/mL
Serum 1,25-Hydroxyvitamin D	15 pg/mL	N/A	25 pg/mL	18-64 pg/mL
Fractional Excretion of Phosphate	93%	N/A	12%	<20 %
Urine Fibroblast Growth Factor-23	264 RU/mL	N/A	103 RU/mL	<180 RU/mL

Oral iron supplementation led to gastrointestinal adverse effects. However, since his hemoglobin remained in the range of 11-12 g/dL and he demonstrated no symptoms of anemia, the decision was made not to administer further IV iron and monitor hemoglobin serially. 

## Discussion

Iron deficiency is the most common nutritional deficiency globally, affecting over 15% of the population [1]. Its prevalence is even higher among individuals with chronic illnesses, menstruating women, and patients with gastrointestinal (GI) disorders. Intravenous (IV) iron therapy is widely used to manage iron deficiency anemia, particularly in patients who are intolerant to oral iron, have poor gastrointestinal absorption, or require rapid replenishment of iron stores [1]. Patients with malabsorptive GI conditions such as celiac disease, post-gastric bypass surgery, inflammatory bowel disease (IBD), and protein-losing enteropathy (PLE) often require IV iron [1]. Intestinal iron absorption is limited: only 20% to 25% of a 100 mg oral iron dose is absorbed, and this maximum rate is typically reached only in the later stages of iron deficiency [1].

The latest generation of IV iron formulations includes ferric carboxymaltose (FCM), ferric derisomaltose (FDI, formerly known as iron isomaltoside), and ferrumoxytol [2,3]. These formulations enable rapid correction of total iron deficit, often in one or two infusions for most patients. Although generally well tolerated, they can cause minor infusion reactions, such as mild arthralgia/myalgia (especially in the chest or flank), facial flushing, hypotension, headache, dizziness, and nausea. However, IV iron-induced hypophosphatemia (IIH) remains an underrecognized yet potentially serious adverse effect [2,3]. Hypophosphatemia has been reported more frequently with FCM and FDI, and only rarely with ferrumoxytol [2,3].

One clinical trial involving 156 IBD patients reported hypophosphatemia in 8.3% of those treated with FDI and in 51% of those treated with FCM [4]. Although previously considered transient, hypophosphatemia can persist for up to six months in about 5% of patients [5,6]. Symptoms can range from mild to moderate, such as fatigue, bone pain, and muscle weakness, which may mimic symptoms of iron deficiency anemia (IDA), to more severe manifestations, including asthenia, myopathy, respiratory failure, and skeletal complications [5,6].

Extensive research into IIH pathophysiology points to increased urinary phosphate excretion and a three- to six-fold rise in fibroblast growth factor 23 (FGF-23), typically occurring within the first 24 hours after FCM infusion [5]. Elevated FGF-23 reduces renal expression of sodium-dependent phosphate co-transporters and suppresses 1-alpha hydroxylase activity in the kidney, leading to decreased synthesis of active 1,25-dihydroxyvitamin D [5]. This cascade results in hyperphosphaturic hypophosphatemia, hypocalcemia, and secondary hyperparathyroidism [2,5]. Collectively, this has been termed the “6H Syndrome”: 1. High FGF-23; 2. Hyperphosphaturia; 3. Hypophosphatemia; 4. Hypovitaminosis D; 5. Hypocalcemia; 6. Secondary hyperparathyroidism [6].

While the 6H Syndrome provides a mechanistic framework for FCM-induced hypophosphatemia, susceptibility to this condition varies. Several risk factors have been identified, including baseline low serum phosphate, vitamin D deficiency, high cumulative or repeated FCM dosing, and preserved renal function (which permits unopposed phosphaturia in response to FGF23) [5]. Other contributing factors include malnutrition, chronic alcohol use, hyperparathyroidism, and a history of bariatric or other malabsorptive GI surgeries [5]. 

There is currently no standardized treatment for IIH, and it often proves refractory to oral and IV phosphate supplementation, as seen in our case. For asymptomatic or mild hypophosphatemia, observation may suffice. However, in cases of significant phosphate depletion or symptomatic patients (e.g., those with bone pain or muscle weakness), management should prioritize discontinuation of FCM, phosphate replacement, and vitamin D supplementation. In our patient, serum vitamin D levels were normal, urinary FGF-23 was elevated, and 1,25-dihydroxyvitamin D was low-findings consistent with IIH. Calcitriol administration resulted in rapid and sustained improvements in serum phosphate levels. This highlights the importance of targeting the underlying pathophysiology when no established guidelines exist. Calcitriol has also been successfully used in previously reported cases of IIH [7]. It is particularly advantageous in malabsorptive conditions like PLE, as it is absorbed via the portal circulation and does not require bile salts or micelle formation for absorption.

## Conclusions

This case demonstrates how ferric carboxymaltose can precipitate profound and persistent hypophosphatemia through FGF-23-mediated phosphate wasting, even in a patient with long-standing nutritional support and stable TPN. The biochemical profile of hypophosphatemia, hypocalcemia, suppressed 1,25-dihydroxy vitamin D, and secondary hyperparathyroidism was highly suggestive of this mechanism, and the failure of conventional supplementation underscored the inadequacy of replacement strategies that do not address the underlying pathophysiology. The patient’s prompt response to calcitriol highlights the value of targeted therapy to restore calcium-phosphate homeostasis and prevent complications such as bone pain, weakness, or fracture risk.

Given the expanding use of intravenous iron formulations, clinicians should be aware that FCM carries a higher risk of this complication than other preparations, and that hypophosphatemia may persist for months rather than resolve spontaneously. Early recognition, electrolyte monitoring, and judicious choice of iron formulation are key, especially in patients with malabsorptive gastrointestinal disease or other risk factors.
